# Meningeal tuberculoma: when to consider it?

**DOI:** 10.1590/0037-8682-0793-2020

**Published:** 2021-03-08

**Authors:** Matheus Augusto Pinto Kitamura, Marcelo Palmares Oliveira e Silva, Vitor Palmares Oliveira e Silva

**Affiliations:** 1 Universidade Federal de Pernambuco, Hospital das Clínicas, Departamento de Neurocirurgia, Recife, PE, Brasil.; 2 Real Hospital Português de Beneficência, Departamento de Neurocirurgia, Recife, PE, Brasil.; 3 Universidade Federal de Pernambuco, Centro de Ciências Médicas, Recife, PE, Brasil.

**FIGURE 1:**
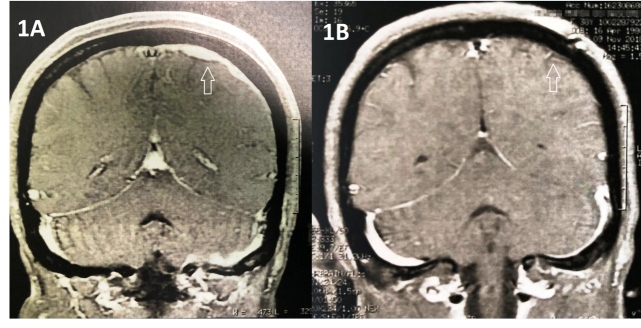
Coronal section; **(A)** Coronal section showing contrast-enhanced meningeal thickening in the upper left hemisphere on T1-weighted MRI; **(B)** Control MRI, after clinical treatment, showing radiological improvement in the meningeal thickening.

**FIGURE 2:**
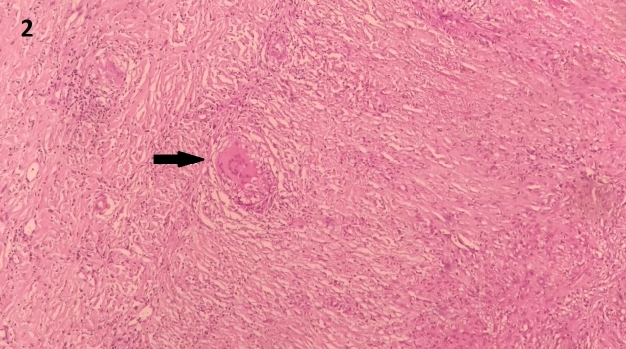
Histological section of the biopsied lesion stained with hematoxylin and eosin showing a caseous granuloma and a central giant cell of the Langhans type (black arrow).

A 38-year-old immunocompetent woman presented with complaints of hemicranial headache and painful ophthalmoplegia, both of which had been persistent and progressive for two years. Fever and other symptoms were not observed. She had a history of tuberculous lymphadenitis that was successfully treated 20 years ago. Contrast-enhanced axial T1-weighted magnetic resonance imaging (MRI) of the brain revealed contrast-enhanced meningeal thickening in the upper left (Figure 1A, white arrow) and left cerebellar hemispheres. Meningioma was the main diagnosis as an investigation of the cerebrospinal fluid returned negative results for the GeneXpert test. However, a histopathological diagnosis of tuberculosis (TB) was made on the basis of a biopsy (Figure 2). Therefore, the patient was initiated on presumptive antituberculosis treatment for 12 months. Further, late MRI demonstrated a radiological improvement in the meningeal thickening (Figure 1B, white arrow).

Central nervous system TB can be classified into four clinical categories in the descending order of incidence: tuberculous meningitis, cerebral tuberculoma, cerebral abscess, and spinal arachnoiditis^1^. TB-mimicking meningiomas are unusual, and their exact representation in MRI remains indefinite^2^. Clinical history or a hypointense pattern in T2-weighted images should draw attention for the diagnosis of tuberculoma^3^.

This report emphasizes the importance of considering cerebral tuberculoma as a differential diagnosis for expansive lesions of the cerebral meninges, especially in endemic regions.
